# Breeding future crops to feed the world through de novo domestication

**DOI:** 10.1038/s41467-022-28732-8

**Published:** 2022-03-04

**Authors:** Hong Yu, Jiayang Li

**Affiliations:** 1 State Key Laboratory of Plant Genomics, and National Center for Plant Gene Research, Institute of Genetics and Developmental Biology, Innovation Academy for Seed Design, Chinese Academy of Sciences, Beijing, 100101 China; 2 University of Chinese Academy of Sciences, Beijing, 100049 China

**Keywords:** Agricultural genetics, Plant breeding, Agricultural genetics, Plant domestication

## Abstract

By the end of this century, a 50% increase in agricultural productivity is required to feed the world. Recent studies have demonstrated de novo domestication of wild plants as a new crop breeding strategy to meet future food challenges.

## Crop domestication and de novo domestication

**Fig. 1:**
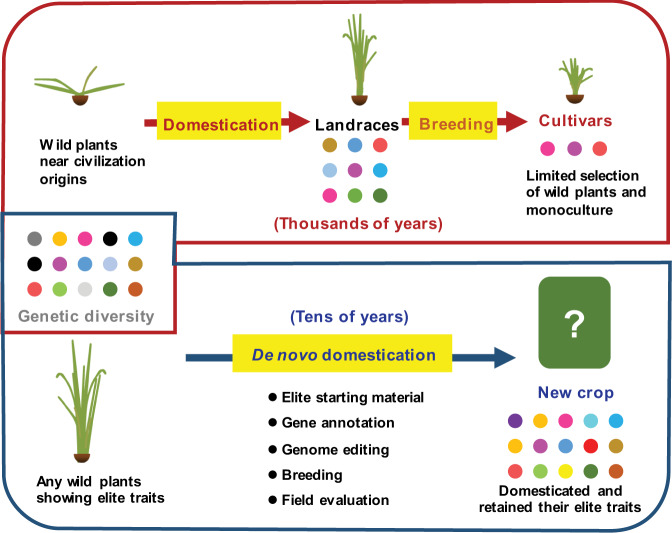
De novo domestication as a promising route in creating future crops. Crop domestication and breeding are critical for the survival and development of human civilization, which cost thousands of years to change the wild plants in civilization origins to modern cultivars; however, the genetic diversity, represented by different color of dots, is greatly reduced due to the intensive selection of wild plants and monoculture of elite varieties. With the technology revolutions, de novo domestication has been proposed and practiced to meet future agricultural challenges, which can speedily select and domesticate elite wild plants while retaining the genetic diversity and the associated elite traits.

Crop domestication was accomplished by the long-term artificial selection, which resulted in the accumulation of beneficial mutations in various landraces and cultivars that fit the cultivation requirements such as less seed shattering, self-pollination, erect growth, shorter awn, and larger seeds. Nowadays, with the development of new biotechnologies especially genome editing, de novo domestication has been proposed as a novel strategy for crop breeding^1,2^. In principle, the de novo domestication strategy starts from selecting the elite foundation materials from wild or semi-wild plant species in nature to meet our new breeding objectives, follows by the rapid introduction of domestication-related traits into them by genetic and breeding tools while retaining their desired features, and ends by creating new crops that harbor beneficial traits compared with current cultivars (Fig. 1).

## New challenges lead to the shift of breeding objectives

After the Industrial Revolution, crop breeding objectives were greatly changed. Crops were supplied or managed with a large amount of fossil-fueled products, including fertilizer and pesticide, freshwater from irrigation systems, and farm machinery. Thus, the breeding objectives were shifted to develop high-yield varieties to adapt to new farming systems. One example was the Green Revolution, which was represented by the development of semi-dwarf wheat and rice varieties that were capable of responding to fertilizer and irrigation without lodging. Between 1950 and 1984, as the Green Revolution updated the global agriculture system, world grain production increased by 250%, but the energy input increased by ~50 times^3^.

However, future agriculture is still facing great challenges^4^. First, the world population is estimated to reach 10–12 billion by the end of this century^5^, requiring at least 50% increase in total food productivity, but the use of fossil fuel is likely to be greatly restricted before that^6^. Second, the world is facing a rapid climate change and more frequent extreme weather conditions, which have become major threatens to agriculture. What are the new strategies to further increase crop yield? How to create crops that are highly resistant to extreme weather conditions and capable of rapid adaptation to climate changes? Answers to these questions are urgently needed^7,8^.

## Efficiency of crop genetic improvement

Breeding has always been an art of science^9^, since it strives to find the best answer in unlimited solution spaces with limited trials. Therefore, the essence of every breakthrough in breeding technology is to elevate the efficiency of crop genetic improvement, so does de novo domestication (Table 1). Hybridization, mutagenesis, transgenics, and genome editing have shown enormous advantages in different aspects of genetic improvement^10^. Hybridization can integrate tens of thousands genes from different parents through genetic recombination, but its thereafter selections require screening large populations in multiple generations. The breeding-by-design strategy has greatly improved the selection efficiency by monitoring the genotypes^11^, but usually only a small number of genes could be monitored and integrated at one time. Mutagenesis can create novel alleles caused by random mutations, however, based on current knowledge and technologies, it is very difficult, if not impossible, to predict the outcomes of random mutagenesis. Although scientific tools were used to drive the creation of new varieties during mutation and recombination, their occurrences are still random and hard to control^12^. In contrast, transgenics can deliver genes of interest into a genome, and genome editing is able to precisely modify the target DNA in the genome and increasingly allows us to make very targeted, specific, and predictable changes. Thus, the outcomes of these two technologies are predictable. However, owing to the limitation of throughput and the cost of genome editing and transgenics, the priority needs to be given to genes determining agronomically important traits.

**Table 1:** Comparison of different plant breeding strategies.

Breeding technologies	Unique features	Advantages	Disadvantages
Hybridization	Integrate genes from different parents	Affecting a large number of genes	Random and laborious in subsequent selections
Mutagenesis	Randomly create new alleles	Creating new alleles	Random and unpredictable
Transgenics	Add genes	Targeted and predictable	Affecting a small number of genes
Genome editing	Make specific changes to DNA	Targeted and predictable	Affecting a small number of genes
De novo domestication	Set new genetic backgrounds	Utilization of genes from wild plants	Requiring genome editing system in wild species

Choices of crop domestication by our ancestors were made by accidental discovery of plants with domestication phenotypes, such as wheat (*Triticum aestivum*) in Europe and Asia, rice (*Oryza sativa*) in China, and Southeast Asia, and corn (*Zea mays*) in Mesoamerica. With the fast development of technologies, it becomes feasible for us to select the best starting materials from all wild plants in nature, which could utilize wild relatives of current crops and extend the choice of crops by examining the new species, referring to re-domestication of crop wild relatives and de novo domestication of new wild species, respectively. In addition, de novo domestication strategy requires the integration of a limited number of genes and thus it is more efficient than the other genetic improvement strategies, as it is a knowledge-based breeding^13^. Many target genes of de novo domestication are the well-studied key domestication-related genes, rather than those beneficial but difficult-to-be-cloned ones. Comparing to the beneficial genes with unknown functions that are targeted by the other genetic improvement strategies, the number of domestication-related genes is much fewer. As generally agreed, the de novo domestication strategy can be divided into four steps: (1) selecting an appropriate starting material, (2) establishing an efficient transformation system and an annotated reference genome, (3) editing of domestication-related genes followed by breeding and field evaluation, and (4) cultivar registration^14^.

The route towards de novo domestication seems straightforward, however, each or all of the above four steps could be technical bottleneck for wild species and even some cultivated crops^15^. Taking soybean as an example, the transformation and genome editing systems are still very hard to establish or very low in efficiency. For many wild species, an annotated reference genome, knowledge of domestication-related genes, and/or a powerful genome editing system are still lacking.

## Demonstration of de novo domestication strategy

Recently, de novo domestication has been successfully demonstrated in multiple species. One example is to retrieve ‘old elite genes’ by domestication of wild relatives of cultivars. As the breeding objectives were shifted from time to time during past agricultural practices, only those objectives-targeted beneficial alleles were selected, while the others, which were thought to be useless at that time, were lost. Therefore, scientists and breeders have attempted to retrieve beneficial alleles from wild species for re-wild breeding^16^. However, these genes, such as disease-resistant genes, are controlled by multiple loci and hard to be cloned, making them inefficient by hybridization or genome editing. In contrast, using CRISPR-Cas9, domestication-related traits were successfully introduced into stress-tolerant wild tomato accessions (*Solanum pimpinellifolium*)^17,18^, which partially achieved the goal of re-wild breeding with high efficiency. Another example shows that groundcherry (*Physalis pruinosa*), an orphan crop distantly related to tomato, could be rapidly improved for increased yield through editing the orthologues of tomato domestication-related genes^19^.

Most recently, we reported the de novo domestication of wild allotetraploid rice (*O. alta*)^20^, which was intended to utilize the advantages of polyploid plants in large biomass, heterosis, genome buffering, and rapid adaptation to climate changes compared to diploid cultivated rice. The transformation and genome editing system was firstly established with high efficiency in *O. alta*. Then, the agronomically important genes in *O. alta* were annotated based on high-quality genome assembly and the knowledge of diploid rice domestication. The genome-edited plants have illustrated that the domestication-related traits could be rapidly modified. We have obtained different edited lines with various improvements in important agronomic traits including plant height, awnless, seed shattering, stem thickness, or heading date. The study illustrates a path to create a novel cultivar that retains the advantages of wild allotetraploid rice and shows higher potentials in grain yield and better adaptation to environmental stresses than current cultivated diploid varieties.

Besides wild tomato, groundcherry, and allotetraploid rice, many wild species with unique advantages may also fit the criteria as appropriate starting materials for de novo domestication, such as quinoa, rubber-producing plants, and wild relatives of kiwi, potato, pepper, and other crops. However, tremendous efforts remain to be taken to fully validate whether novel elite crops could be created. The complete domestication of wild plants requires at least tens of edited alleles/genes with variant modifications, as many domestication and improvement genes are expression variants. Although de novo domestication is much more efficient than traditional breeding as illustrated above, the screening and phenotypic evaluation of different edited alleles, large-scale multiplex editing and target genotyping, and the pyramiding of tens of edited genes are laborious and costly. The time of creating real crop with good performance in field conditions may range from years to decades, depending on the number of genes to be edited and the growth cycle of wild plants. Considering that biotechnologies are developing very rapidly, it is very likely that these bottlenecks will be overcome in the very near future. We are optimistic that similar strategies will be applied to other crop species, which may lead to a new era of crop breeding.
